# Adipose-derived mesenchymal stem cells from patients with atherosclerotic renovascular disease have increased DNA damage and reduced angiogenesis that can be modified by hypoxia

**DOI:** 10.1186/s13287-016-0389-x

**Published:** 2016-09-09

**Authors:** Ahmed Saad, Xiang-Yang Zhu, Sandra Herrmann, LaTonya Hickson, Hui Tang, Allan B. Dietz, Andre J. van Wijnen, Lilach Lerman, Stephen Textor

**Affiliations:** 1 Division of Nephrology and Hypertension, Mayo Clinic, Rochester Minnesota, 200 First Street SW, Rochester, MN USA; 2 Division of Transfusion Medicine, Mayo Clinic, Rochester Minnesota, 200 First Street SW, Rochester, MN USA; 3 Department of Orthopedic Surgery, Biochemistry and Molecular Biology, Mayo Clinic, Rochester Minnesota, 200 First Street SW, Rochester, MN USA

**Keywords:** Mesenchymal stem cells, Hypoxia, Renovascular disease, Angiogenesis, VEGF and MicroRNAs

## Abstract

**Background:**

Adipose-derived MSC (AMSCs) possess angiogenic and immunomodulatory properties that may modulate kidney regeneration. Whether these properties are retained in older patients with atherosclerotic vascular disease is poorly understood. Hypoxic conditions are known to modify properties and growth characteristics of AMSCs. We tested the hypothesis that AMSCs from older patients with atherosclerotic renovascular disease (RVD) differ from normal kidney donors, and whether hypoxia changes their functional and molecular properties to promote angiogenesis.

**Methods:**

AMSCs from 11 patients with RVD (mean age =74.5 years) and 10 healthy kidney donors (mean age = 51.2 years) were cultured under normoxia (20 % O_2_) and hypoxia (1 % O_2_) for 3–4 days until they reached 80 % confluency. We analyzed expression of genes and microRNAs using RNA sequencing and real-time quantitative rt-PCR. Protein expression of selected angiogenic factors (VEGF, IGF, HGF and EGF) were quantified in conditioned media using ELISAs. Apoptosis was tested using Annexin IV staining.

**Results:**

Normoxic AMSC from RVD patients grew normally, but exhibited increased DNA damage and reduced migration. VEGF protein secretion was significantly lower in the RVD AMSCs (0.08 vs 2.4 ng/mL/ cell, *p* <0.05) while HGF was higher. Both trends were reversed during growth under hypoxic conditions. Hypoxia upregulated pro-angiogenic mRNAs expression in AMSCs (VEGF, FGF, STC and ANGPTL4), and downregulated expression of many miRNAs (e.g., miR-15a, miR-16, miR-93, miR-424, 126, 132, 221) except miR-210.

**Conclusions:**

Thus, although AMSC from patients with RVD had increased DNA damage and reduced migration, hypoxia stimulated pro-angiogenic responses via increased expression of angiogenic genes, VEGF secretion and induction of the hypoxia-inducible miR-210, while downregulating angiogenesis-related miRNAs.

**Electronic supplementary material:**

The online version of this article (doi:10.1186/s13287-016-0389-x) contains supplementary material, which is available to authorized users.

## Background

Occlusive atherosclerotic renovascular disease (RVD) reduces renal blood flow (RBF), eventually producing renal ischemia, tissue hypoxia, and oxidative stress [1, 2]. These changes lead eventually to microvascular dysfunction, activation of inflammatory pathways, and fibrosis [3, 4]. In experimental models, therapies based on adipose-derived mesenchymal stem/stromal cells (AMSCs) enhance microvascular growth and enhance kidney repair and functional recovery, particularly when combined with renal revascularization. Administration of AMSCs in the swine model of RVD is associated with increased vascular endothelial growth factor (VEGF) and suppression of inflammatory cytokines. These changes are accompanied by increased RBF and glomerular filtration rate (GFR), and reduced fibrosis in the post-stenotic kidney [5]. Although the exact mechanisms have not yet been elucidated, regenerative characteristics of MSCs are widely attributed to paracrine effects mediated through secretion of bioactive molecules and chemokines [6].

Potential barriers to clinical application of MSC-based therapy in the kidney include the likelihood for reduced functional capacity related to age, atherosclerotic disease, impaired kidney function and the milieu of diseased tissue. Stem cells are prepared and maintained in vitro with favorable growth conditions and abundant oxygen levels. However, the injured tissue milieu in vivo and surrounding the cells are often hypoxic, have increased levels of inflammatory mediators, and low pH. These parameters collectively may affect the efficacy of cell-based therapy [7–9]. MSC used presently for regenerative medical purposes potentially may undergo apoptosis prior to differentiation into the desired cell type due to these nonideal environmental conditions [10]. Aging, for example, is associated with a progressive decrease in the angiogenic capacities and regenerative potential of MSCs [11].

Strategies designed to modify the functional properties of MSCs may enhance specific features including survival, angiogenesis and anti-inflammatory activity. Through the manipulation of specific genetic targets, many cellular processes can either be enhanced or suppressed. These changes can give the stem cell a powerful advantage in the ability to survive in the face of hostile conditions within the tissue. One approach to improving efficacy of MSCs has been to grow them under hypoxic conditions mimicking the in vivo environment in ischemic tissues like RVD in which oxygen tensions may be 1 % or lower. When cultured under ‘hypoxic’ conditions (typically 1–4 % oxygen), MSCs demonstrate upregulation of numerous functions, including growth factor receptors and migratory capacity [12]. When infused into acutely infarcted heart experimentally, hypoxic preconditioned MSCs perform better than unconditioned control cells leading to improved cardiac function and reduced infarct size [13, 14]. However, the exact mechanisms by which hypoxia exerts its effects remain unclear and whether hypoxic conditions modify AMSCs from elderly patients with atherosclerosis are poorly understood.

The purpose of this study was to examine the differences between AMSCs from RVD patients compared to healthy subjects (normal kidney donors) and the effects of hypoxia on the functional properties of AMSCs, including survival and angiogenic capacity. Our hypothesis was that functional characteristics of AMSCs from patients with RVD including angiogenic, protein expression, and paracrine effects differ from healthy individuals. We further hypothesized that hypoxic growth conditions could modify the angiogenic and functional characteristics of AMSCs from RVD patients. We addressed these two hypotheses by evaluating the biological properties of AMSCs, as well as by comparing expression of protein-coding mRNAs and regulatory noncoding microRNAs (miRNAs) under normal and hypoxic conditions.

## Methods

### Patients

AMSCs were obtained from patients with atherosclerotic RVD (*n* = 11) and healthy volunteers (*n* = 10) (Table 1). The RVD patients were enrolled for autologous AMSCs infusion as a part of separate treatment protocol under an Investigational New Drug study (FDA 15082, clinicaltrials.gov). Inclusion criteria for RVD were ages between 40 and 80 years, advanced vascular occlusive disease affecting one or both kidneys defined as loss of parenchymal volume and severe renal artery stenosis measured by duplex ultrasound velocity above 300 cm/sec to the affected kidney and serum creatinine below 2.5 mg/dL. The healthy volunteers were kidney donors undergoing laparoscopic donor nephrectomy with age ranging from 20 to 40 years, as young healthy and another group from 40 to 80 years old considered as old healthy control, all of them without chronic health problems.

**Table 1:** Clinical and demographic data of Donors and RVD patients

	AMSCs (healthy) *N* = 10	AMSCs (RVD) *N* = 11	*p* value
Age	51.2 ± 16.7	74.5 ± 3.9	=0.009
Gender (female/male)^a^	6/4	4/7	<0.0001
Creatinne (mg/dL)	0.92 ± 0.13	1.48 ± 0.29	<0.0001
eGFR mL/min	77.1 ± 12.7	45.2 ± 9.9	<0.0001

*AMSCs* adipose-derived mesenchymal stem/stromal cells, *RVD* renovascular disease, *eGFR* estimated glomerular filtration rate

^a^Fisher’s exact test

### Isolation and culture of AMSCs

A subcutaneous fat biopsy (approximately 2–4 g) was obtained under sterile conditions from RVD patients in an outpatient surgical suite or during the surgical nephrectomy from kidney donors. Cells were expanded ex vivo as follows. The fat tissues were minced with surgical scalpels and incubated in 2 % collagenase type H (Roche, Mannheim, Germany) for 90 min at 37 °C. Digested tissue was centrifuged at 400 g for 5 min with the pellet washed in PBS, passed through a 70-μm cell strainer (BD Biosciences, San Jose, CA, USA), and incubated in red blood cell lysis buffer (154 mM NH4Cl, 10 mM KHCO3, 0.1 mM EDTA). Cells were grown in T-75 cm^2^ flasks at a concentration of 1.0–2.5 × 10^3^ cells/cm^2^ in Advanced MEM with 5 % PLTmax (Mill Creek Life Sciences, Rochester, MN, USA) and 2 mM L-glutamine (Invitrogen, Carlsbad, CA, USA) in a 37 °C 5 % CO^2^ incubator for 3–4 days. When cells were 60–80 % confluent, they were passaged using TrypLE (Trypsin-like Enzyme, Invitrogen) [15]. Cell yield was quantified with a Countess hemocytometer (Thermo Fisher Scientific, Inc., NY, USA).

### Experimental design of the hypoxia protocol

Cultured AMSCs were maintained (passage 3–5) under normoxic (20 % O_2_) or hypoxic conditions (1 % O_2_) for 3–5 days. Hypoxia was achieved by placing cells in a Modular Incubator Chamber (Billumps-Rothenberg; Del Mar, CA, USA) that was flushed with a mixture of 1 % O_2_, 5 % CO_2_, and 94 % N_2_, confirmed by an infrared gas analyzer (Novametrics, Wallingford, CT, USA).

### Flow cytometry characterization of AMSCs

Isolated AMSCs were characterized by immunostaining and fluorescence-activated cell sorting (FACS) analysis to determine cellular phenotype for the MSC markers CD34, CD14, CD45, CD44, CD73, CD90, CD105 (1:100; Abcam, Cambridge, MA, USA and BD Pharmigen, San Jose, CA, USA). Population-doubling time (PDT) was computed by linear regression of log2 values of cell number.

### High-throughput RNA sequencing

Total RNA was isolated from confluent AMSCs of three RVD patients (RVD-1, -2 and -4) and three healthy controls (Healthy-1, -5 and -7) both under normoxia and hypoxia, to characterize the basal state of expression by high-throughput RNA sequencing using the TruSeq method (“poly A RNA Seq”) as previously described [16]. RNA libraries were prepared according to the manufacturer's instructions for the TruSeq RNA Sample Prep Kit v2 (Illumina, San Diego, CA, USA). The miRNA-Seq data were analyzed using CAP-miRSeq v1.1 [17]. Normalization and differential expression analysis were performed using edgeR 2.6.2 [18].

### mRNA expression analysis

Expression values for each gene were normalized to 1 million reads and corrected for gene length (reads per kilo base pair per million mapped reads, RPKM). Genes with RPKM >0.1, fold change (hypoxia/normoxia) >1.4 and *p* values <0.05 (hypoxia vs. normoxia, Student’s *t* test) were classified as genes enriched in hypoxia [16].

### miRNA expression analysis

miRNA expression levels (normalized total reads) of AMSCs were examined under normoxic and hypoxic conditions, and the fold change enrichment was calculated. We used miRDB (Version 6.2) to predict target genes for miRNA with fold change >1.4 and *p* values <0.05 (Student’s *t* test) [19].

### Measurement of miRNAs by quantitative real-time PCR

To confirm the results from miRNA-Seq, we selected some miRNAs of interest to measure using PCR. Total RNA was isolated from AMSCs by the mirVana PARIS total RNA isolation kit (Life Technologies, Carlsbad, CA, USA, Cat# AM1556) according to the kit protocol. RNA concentrations were measured by a NanoDrop Spectrophotometer (NanoDrop, Thermo Fisher Scientific, Inc.). A fixed volume of 5 μL of RNA elute at 1 ng/uL was reverse transcribed by using the TaqMan MicroRNA Reverse Transcription Kit (Life Technologies, Cat# 4366596). For PCR, 1.33 μL of RT product was combined with 10 μL of TaqMan Universal Master Mix (Cat# 4440038), 7.67 μL of H2O and 1 μL of primers, including miR-21, miR-146a, miR-155, and miR-210 (Life Technologies, Cat# 000397, 001097, 002623, and 000512 respectively) to make up a 20 μL reaction. RNU6B (Life Technology Cat# 001093) was included in the assay as reference control. Real-time PCR was carried out on an Applied Biosystems (Foster City, CA, USA) ViiA7 Real-Time PCR system at 50 °C for 2 min, 95 °C for 10 min and 40 cycles of 95 °C for 15 s and 60 °C for 1 min. Fold changes of miRNA levels in hypoxia relative to normoxia were calculated using the 2^−ΔΔCt^ method.

### ELISA

Conditioned media aliquots were collected before harvesting the cells and stored at −80 °C until they were assayed. Levels of VEGF (Life Technology KHG0111), epidermal growth factor (EGF R&D Systems, Minneapolis, MN, USA, DEG00), hepatocyte growth factor (HGF, R&D Systems, DHG00) and insulin-like growth factor 1 (IGF-1, R&D Systems, DG100) were measured by enzyme-linked immunosorbent assays (ELISAs) according to manufacturer protocols.

### Cell viability, migration, and apoptosis

Cell viability was detected by trypan blue staining [20] that identifies cells in which the cell membrane is compromised. Cells were diluted 1:100 in 0.25 % trypan blue solution (Invitrogen) and counted in a hemocytometer to assess the number of dead blue cells from the total number of cells counted. An additional measure of cell viability was obtained using FACS for Annexin V. After harvesting the AMSCs, cells were centrifuged for 5 min at 1200 rpm.

Apoptotic cells were detected using APC Annexin V/Dead Cell Apoptosis Kit with APC Annexin V and SYTOX® Green (Invitrogen, Eugene, OR, USA). Cells were resuspended at a concentration of 1 × 10^6^ cells/mL in 1X Annexin-binding buffer; 5 μL APC-Annexin V and 1 μL of the 1 μM SYTOX® Green stain working solution were added to each 100 μL cell suspension. The cells were incubated at 37 °C in an atmosphere of 5 % CO2 for 15 min, and then analyzed by flow cytometry [21]. DNA damage that is associated with cellular senescence and migration were each measured by western blot using, respectively, H2AX antibodies that detect nuclear sites with telomere loss or other DNA damage, and the EMD Millipore (Billerica, MA, USA) QCM™ 24-well chemotaxis cell migration assay (Cat# ECM508) as described [22, 23].

### Statistical analysis

Student’s *t* test was used to compare data between the two experimental groups. Genes with RPKM >0.1, fold change (hypoxia/normoxia) >1.4 and *p* values <0.05 (hypoxia vs. normoxia, Student’s *t* test) were classified as genes enriched in hypoxia. MiRDB (Version 6.2) was used to predict target genes of miRNA with fold change >1.4 and *p* values <0.05 (Student’s *t* test). Data are expressed as the mean ± SD. *p* <0.05 was considered statistically significant.

## Results

Patient demographics: data obtained from AMSCs from 11 patients with RVD and 10 healthy volunteers are summarized in Table 1.

### Characteristics of human AMSCs in RVD patients under normoxic conditions

**Fig. 1:**
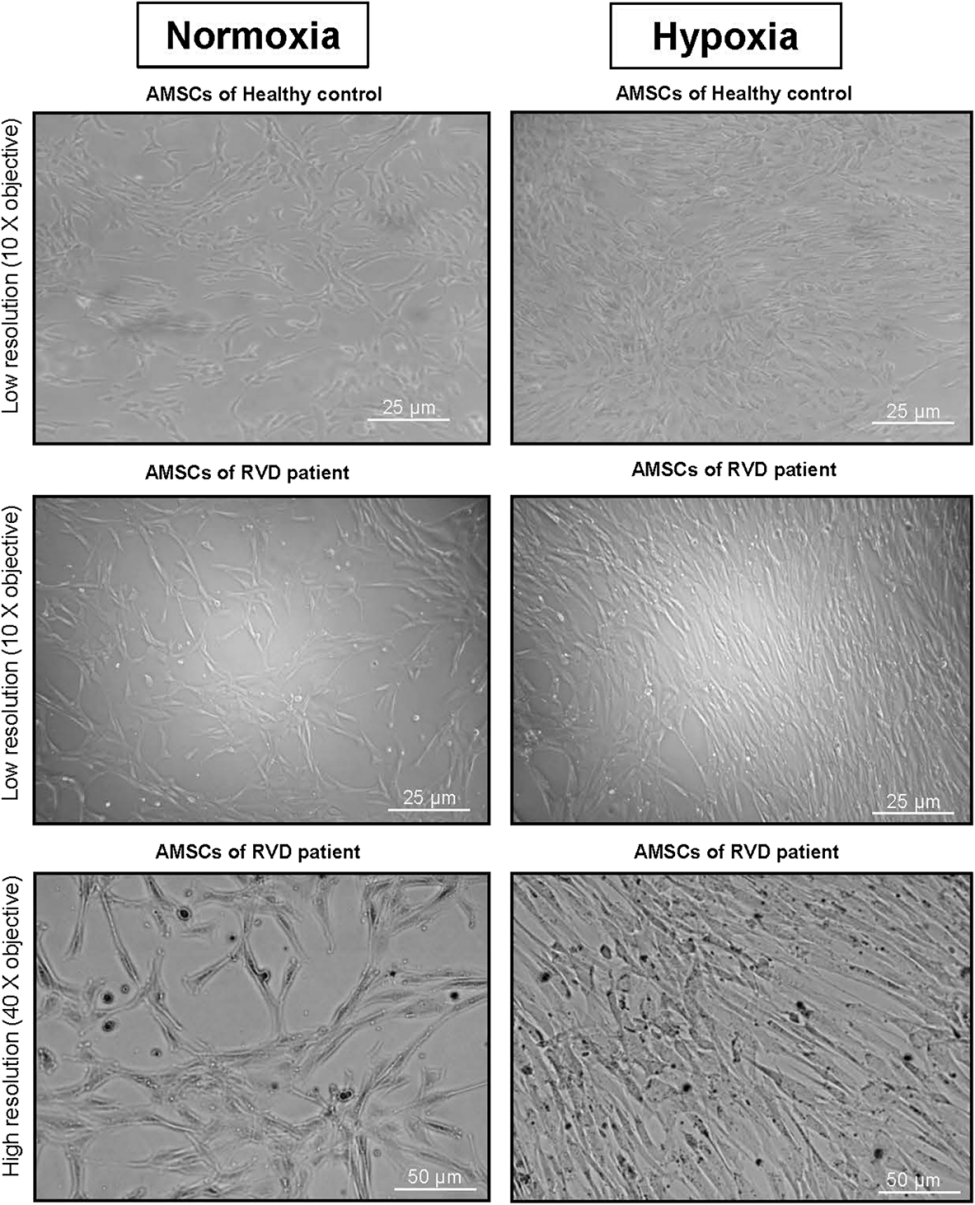
Morphologic appearance of AMSCs grown under normoxic and hypoxic conditions from healthy kidney donors (*top row*) and older individuals with atherosclerotic RVD (*lower rows*). Although growth rates were not changed under hypoxic conditions, the morphological appearance of AMSCs was different after hypoxia compared with normoxia (*left*, normoxia; *right*, hypoxia). The hypoxic AMSCs appeared bigger, denser and more granular (higher magnification - *bottom row*). *AMSCs* adipose-derived mesenchymal stem/stromal cells, *RVD* renovascular disease

**Fig. 2:**
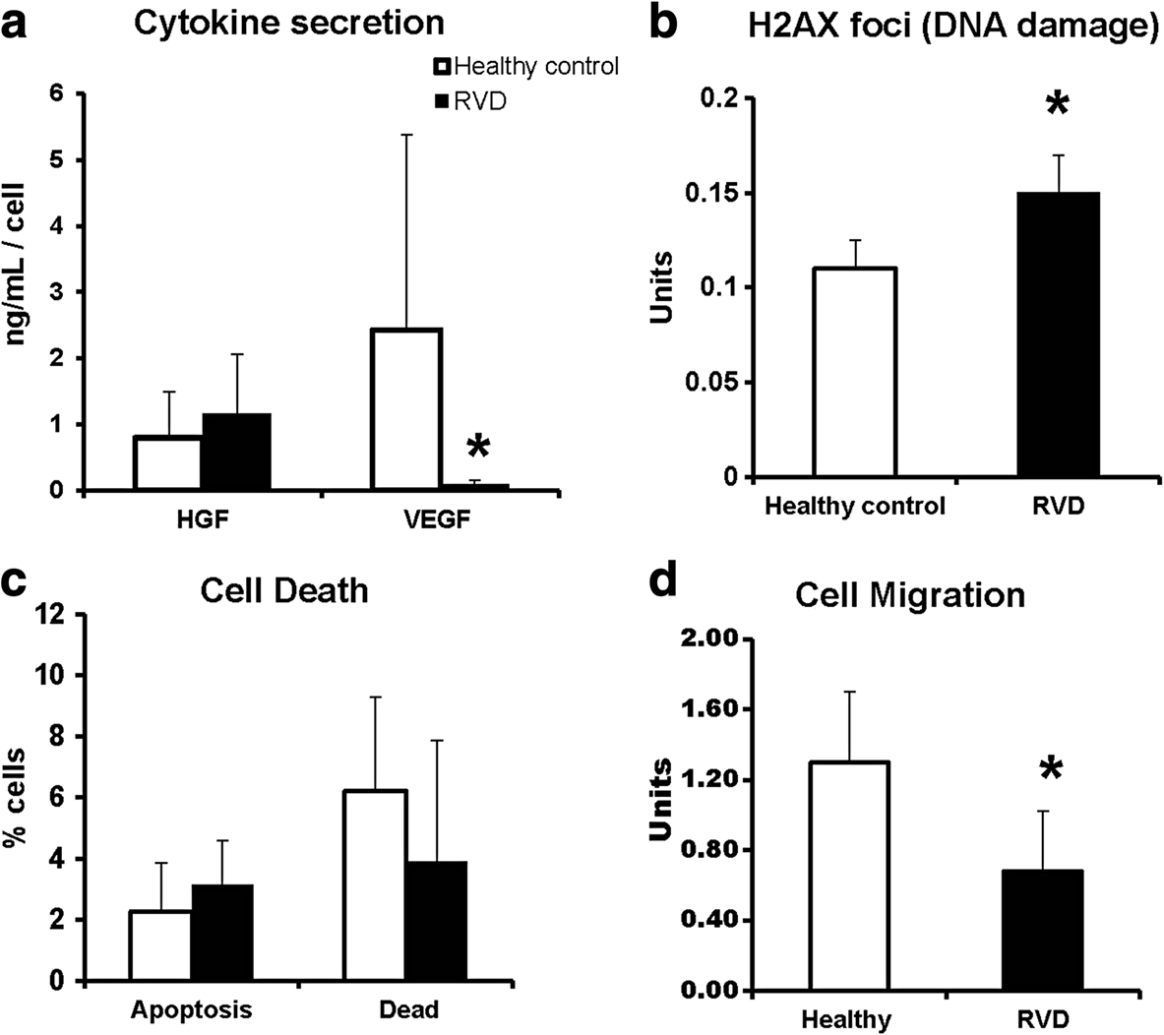
Functional differences between RVD AMSC expression and normal donors: VEGF secretion from AMSCs was low in RVD while HGF secretion is a higher compared to healthy controls (**a**). Senescence-associated DNA damage was increased in RVD patients compared to healthy individuals (**b**). The percentage of apoptosis was similar between RVD and healthy donors (**c**). AMSCS from RVD patient had lower migratory capacity compared to healthy control (**d**). *HGF* hepatocyte growth factor, *RVD* renovascular disease, *VEGF* vascular endothelial growth factor

Human AMSCs from adipose tissue from both healthy and RVD patients retained characteristic plastic-adherent, fibroblast-like morphology, expressed HLA-ABC, CD44, CD90, CD29, CD73, and CD105 markers, and did not express HLA-DR, CD45, CD14 or CD34 markers either under normoxia or hypoxia (Additional file 1: Figure S1). The morphology of cells grown under hypoxia differed slightly in some individuals (both healthy and RVD), insofar as the cells appeared larger and more granular (Fig. 1). VEGF secretion in the supernatant of RVD AMSC at normoxic conditions was markedly lower compared to healthy controls (*p* <0.05), whereas HGF levels were slightly higher (Fig. 2a). These changes were mainly due to age rather than vascular disease, as cytokine secretion (IGF, HGF and EGF) was not different in RVD compared to older healthy subjects except for VEGF (Fig. 7). Compared to healthy volunteers (*p* <0.05), AMSCs from patients with RVD had higher levels of nuclear foci for the phosphorylated form of histone variant H2AX (Fig. 2b). This protein is recruited to sites of DNA damage and phosphorylated by DNA repair kinases, which is particularly evident in aging cells that lose telomeres prior to becoming fully senescent. Under normoxia, cell viability (measured with trypan blue) and percentage of apoptotic cells (measured by Annexin V) were similar in both groups (Fig. 2c). The AMSCs from patients with RVD had lower migration capacity compared to healthy volunteers (Fig. 2d).

### Hypoxia upregulated survival and pro-angiogenic gene expression of RVD AMSCs

**Fig. 3:**
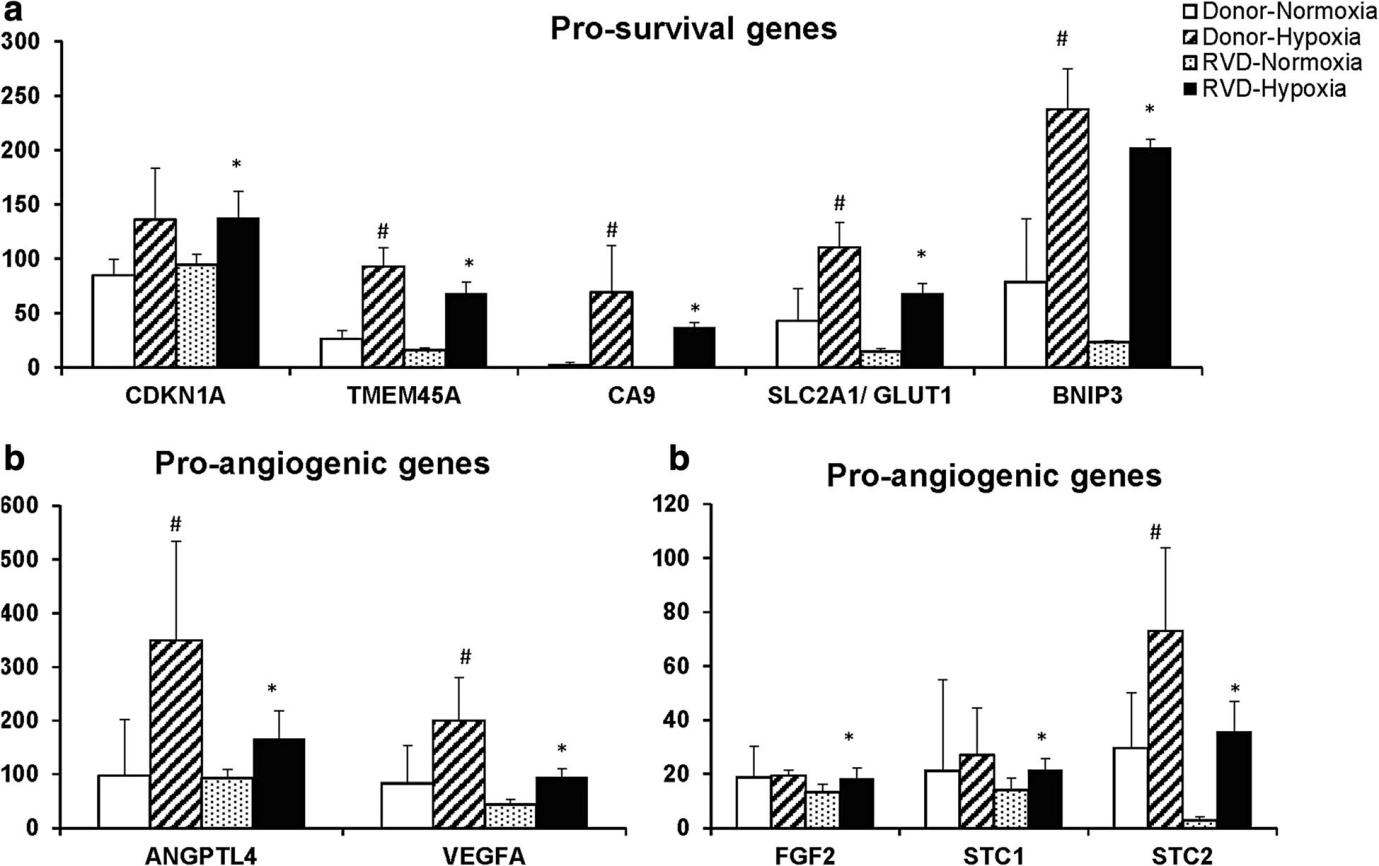
Upregulation of gene expression of AMSCs under hypoxia in both RVD and healthy individuals: hypoxia upregulated pro-survival genes (CDKN1A, HMOX1, TMEM45A, CA9, SLC2A1/ GLUT1, and the autophagy regulatory gene BNIP3) (**a**). Hypoxia upregulated pro-angiogenic genes (ANGPTL4 and VEGF-a) (**b**) and (STC1, STC2, and FGF2) (**c**). *RVD* renovascular disease

Expression of more than 3050 mRNAs (out of 23,398 mRNAs) was different in RVD AMSCs vs. healthy volunteers (fold change >1.4; *p* values <0.05; RVD vs. healthy volunteers). Expression of more than 460 genes with adequate basal levels (RPKM >0.5) was increased under hypoxic conditions (fold change >1.4; *p* values <0.05; hypoxia vs. normoxia) This set of mRNAs encode regulators of metabolism, DNA repair, cell cycle regulation, and proteins involved in related functions associated with cell survival (Additional file 2: Figure S2). Among these were Cyclin-dependent kinase inhibitor 1A (CDKN1A), TMEM45A, Nox4, BNIP3, SLC2A1 or glucose transporter 1 (GLUT1), carbonic anhydrase 9 (CA 9) and heme oxygenase 1 (HMOX1) genes associated with increased cell survival and anti-apoptotic roles that were highly expressed under hypoxia in both healthy and RVD AMSCs (*p* <0.05) (Fig. 3a). Hypoxia decreased the expression of protein tyrosine phosphatase, non-receptor type 2 (PTPN2) that negatively regulates numerous signaling pathways and biological processes including cell proliferation and cell growth. Hypoxia increased the expression of BNIP3 and ATG9b, which are known to induce mitochondrial autophagy as well as the pro-angiogenic genes VEGF, FGF, Stanniocalcin-1, -2 (STC 1, 2) and angiopoietin-like 4 (ANGPTL4) (*p* <0.05) (Fig. 3b,c) both in RVD and healthy donors but to a higher degree in RVD. HGF expression levels were lower under hypoxia in RVD and healthy AMSCs. These data indicate that hypoxia modulates the biological functions of AMSCs and expression of a number of paracrine factors.

### Hypoxia maintained AMSCs cell viability and proliferation without affecting apoptosis

**Fig. 4:**
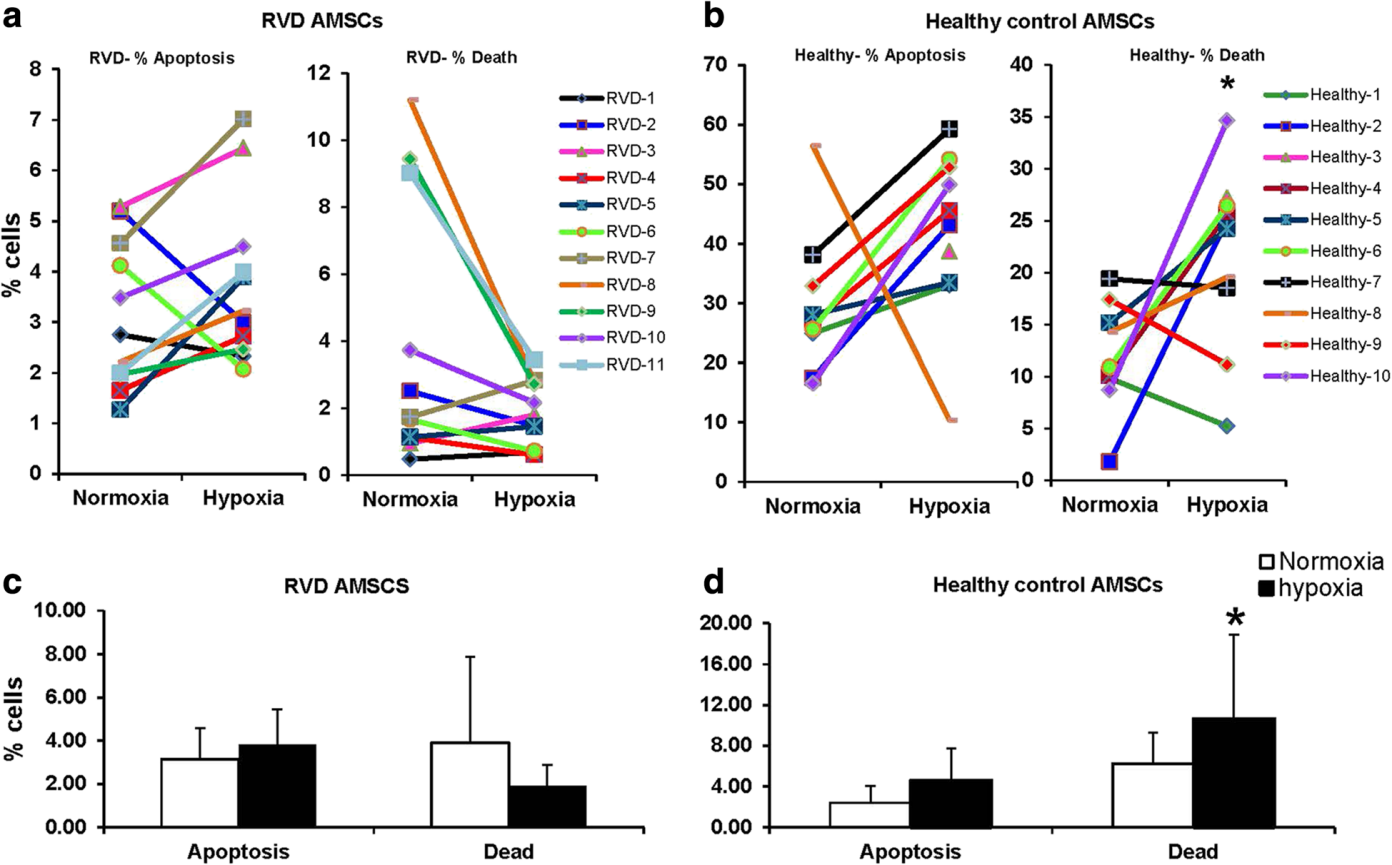
Differential effects of hypoxic conditions on RVD versus normal kidney donors. Graphs showing within-individual differences and changes in percent of apoptosis and death under hypoxia in RVD (**a**) and healthy control (**b**). The percentage of apoptosis (measured by Annexin V) in RVD and healthy controls did not change under hypoxia. However, hypoxia increased cell death levels only in the healthy group (**c** and **d**). AMSCs adipose-derived mesenchymal stem/stromal cells, RVD renovascular disease

Levels of viable cells from donors/RVD patients were above 90 % as measured with trypan blue. Although, the percentage of apoptosis (measured by Annexin V) in both groups did not change with hypoxia, the individual differences between donors and/or RVD AMSCs were significant (Fig. 4). While it was minimal, the differential effects of hypoxia on cell viability for AMSCs from RVD patients indicate that the diseased state may have favored adaptation of AMSCs to low oxygen tolerance.

### miRNAs regulating survival and pro-angiogenic genes were downregulated under hypoxic conditions

**Fig. 5:**
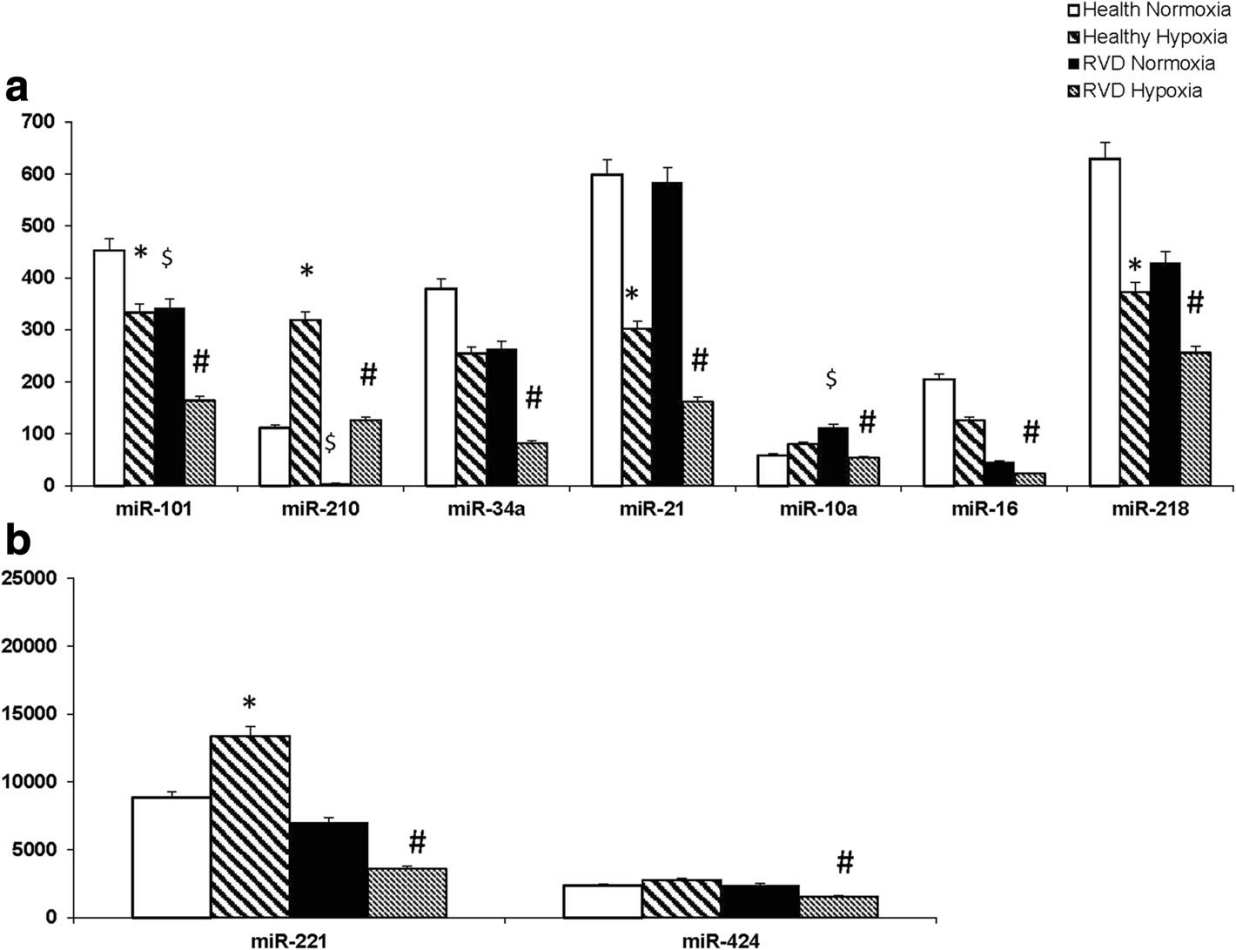
Hypoxia changed the expression of miRNAs regulating survival and pro-angiogenic genes: hypoxia upregulated the pro-angiogenic miRNA-210 (65 FC in RVD, but to a lesser degree in healthy control 2.9 FC) (**a**), whereas downregulated pro-apoptotic miRNAs (miR-34a, miR-15, and miR-16) and also downregulated antiangiogenic miRNAs (miR-221/222 and miR-424) only in RVD (**b**). *AMSCs* adipose-derived mesenchymal stem/stromal cells, *RVD* renovascular disease

There were 415 out of 2253 miRNAs that were different in RVD from healthy donors under normal conditions (FC >1.4). We detected more than 360 miRNAs with acceptable basal levels (normalized reads >0.2) and differentially expressed in hypoxia (fold change >1.4; *p* values <0.05; hypoxia vs. normoxia). The majority of these miRNAs (approximately 85 %) were downregulated under hypoxia, with the most notable exception being the pro-angiogenic miR-210 (that increases VEGF levels), which was highly upregulated (fold change FC >65, in RVD cells). miRNA-210 was also increased in healthy control AMSCs under hypoxia (2.9 FC) but to a lesser degree than was seen in RVD. Baseline miR-210 was low in RVD compared to healthy control AMSCs (Fig. 5a). The miRNAs that exhibited reduced expression under hypoxia, included pro-apoptotic miRNAs (miR-34a, miR-15 and miR-16), senescence-associated miRNAs (miR-10a and miR-21) and anti-angiogenic miRNAs (miR-221/222, miR-24, miR-29, miR-15, miR-16, miR-17, miR-424, miR-20b, miR-218, miR-132, miR-92a, and miR-101) (Fig. 5b). The latter miRNAs may negatively modulate angiogenesis by repressing VEGF expression under normoxic conditions. For example, reduction of miR-15, miR-16, and miR-424 contributes to increased VEGF expression [24–26]. Downregulation of miR-221 correlates with an increase in its target gene expression CDKN1A in our study (Fig. 3a), consistent with previous observations [27]. There were not similar effects of hypoxia on most miRNAs from healthy donors AMSCs ‘both young and old’.

### Hypoxia increased the posttranscriptional protein secretion of VEGF but decreased HGF

**Fig. 6:**
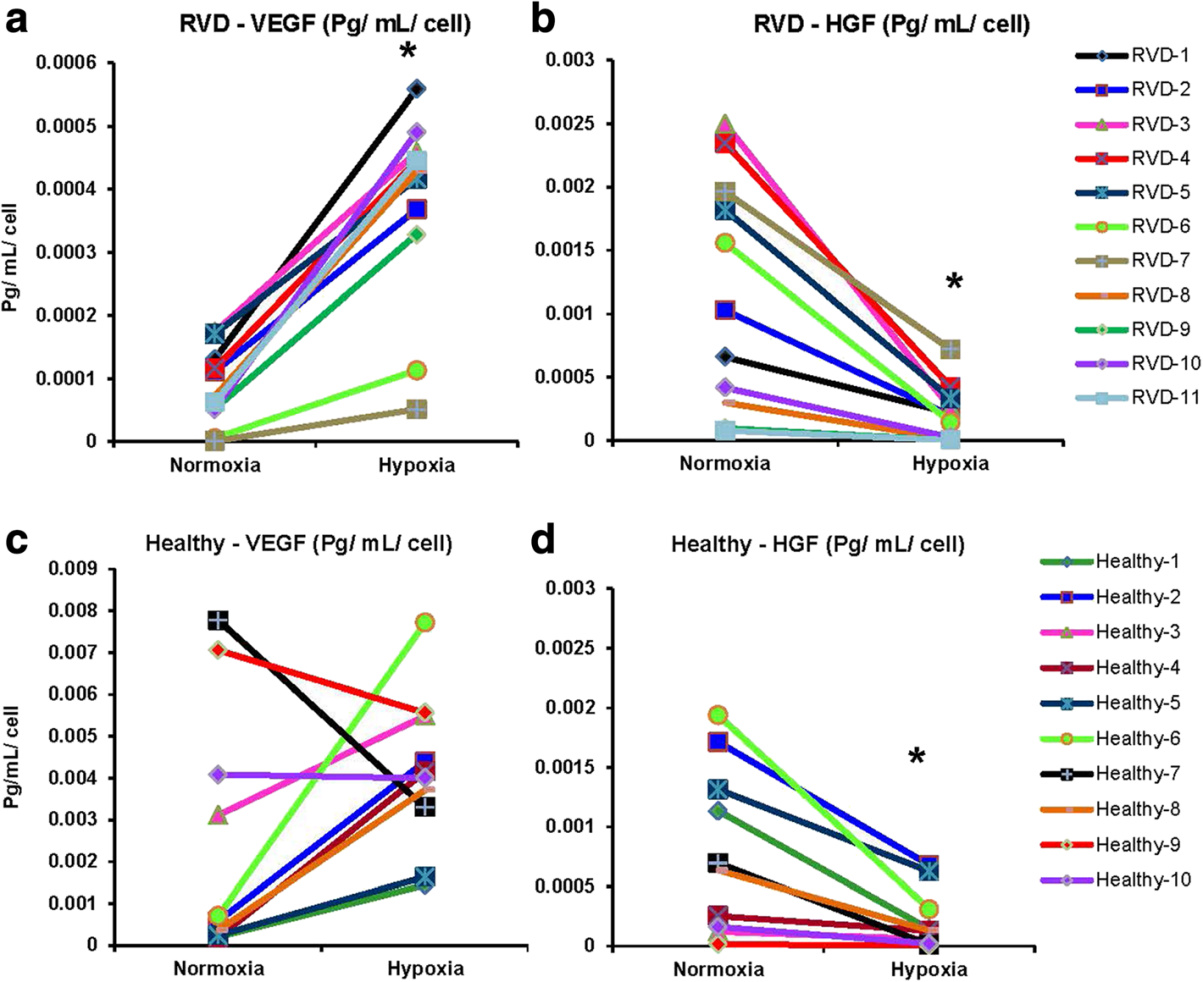
Hypoxia increased the posttranscriptional protein secretion of VEGF (**a**) but decreased protein secretion of HGF (**b**) in RVD AMSCs. In AMSCs from healthy controls, hypoxia did not change VEGF (**c**) but decreased HGF (**d**). Graphs showing within-individual changes in VEGF and HGF secretions under hypoxia in RVD, healthy control. *HGF* hepatocyte growth factor, *RVD* renovascular disease, *VEGF* vascular endothelial growth factor

Hypoxia increased the secretion levels of VEGF in the supernatant of RVD AMSCs (*p* < 0.0001) and AMSCs from old healthy individuals (*p* = 0.007) to levels seen in young healthy cells from donors. There was no such effect of hypoxia in young donor AMSCs (*p* = 0.35). Conversely, hypoxia decreased HGF protein levels in the supernatant both in RVD and healthy donors (Fig. 6), again, this effect was predominately in old individuals (*p* = 0.01 vs. *p* = 0.06, old vs. young). IGF and EGF secretions tended to increase under hypoxia. Hypoxia increased secretions of IGF, EFG, and VEGF and decreased HGF from old healthy AMSCs to normal levels seen in young healthy cells (data not shown).

## Discussion

The results of this study identify considerable differences between AMSCs from patients with atherosclerotic RVD and those from healthy controls. Some of these differences were related to older age, but were magnified by disease. Platelet lysate-expanded AMSC from RVD patients exhibited greater levels of senescence-associated DNA damage and reduced migration capacity as compared to AMSC from healthy individuals. Importantly, expression of angiogenic proteins such as VEGF and EGF was reduced in RVD patients, although of HGF levels were higher. Expansion of AMSC from RVD subjects under hypoxic conditions largely reversed the differences seen under normoxic conditions. Hypoxic conditions resulted in a more robust increase in VEGF, which is a key stimulant for angiogenesis. Senescence-associated DNA damage was increased in elderly patients with RVD compared to healthy individuals. Hypoxia enhanced AMSC survival as reflected by a reduced fraction of dead cells in some patients. The angiogenic functions of RVD AMSCs increased to normal levels during hypoxic expansion as reflected by enhanced expression of angiogenesis-related mRNAs (Fig. 3b), alterations in relevant miRNAs and increased secretion of VEGF, IGF and EGF cytokines.

Hypoxic growth has been employed as a strategy to enhance stem cell in vivo survival and tissue regeneration [10]. Our results indicate that these conditions upregulate angiogenic factors and downregulate apoptotic effector pathways. MicroRNAs regulate cellular functions by translational repression and/or mRNA degradation. We focused on common miRNAs known to regulate survival and angiogenesis and their target gene and posttranscriptional protein expressions. Under hypoxia, miR-210 is most consistently and robustly induced [28]. MiR-210 mediates the proliferation and migration under chronic hypoxia and ultimately leads to downregulation of PTPN2. Consistent with previous studies, which showed that inhibition of PTPN2 (a direct target gene of miR-210) lead to increased proliferation and migration of AMSCs [29], the AMSC from RVD patients under hypoxia in our study highly expressed miR-210 (FC > 65) and downregulated PTPN2.

Our results demonstrate further that hypoxia reduced the expression of miR-34a (FC > -3). Enhanced expression of miR-34a induces apoptosis, cell cycle arrest and differentiation, or reduces migration [30, 31]. Reducing the expression of the pro-apoptotic miR-34a improves survival of bone marrow stem cells in vitro and enhances the therapeutic benefit of cell therapy in mice after acute myocardial infarction [32]. Previous studies have shown that suppression of miR-10a and miR-21 in aged endothelial progenitor cell (EPCs) increase HMGA2 expression, rejuvenate EPCs, resulting in decreased senescence [33]. Hypoxia downregulated both miR-10a (FC > -2) and miR-21 (FC > -3), but without much change in HMGA2 in RVD. CDKN1A is a gene known to regulate cell survival and growth [34] and is regulated by miR-221-3p and miR-93 [35]. Similarly, our results showed that hypoxia downregulated miR-221 (FC > -2) and miR-93 (FC > -2), CDKN1A was upregulated, thereby potentially promoting survival of AMSCs.

Another important gene overexpressed under hypoxia was HMOX1, which is known to have anti-inflammatory effects and may improve survival in the ischemia/reperfusion-acute kidney injury microenvironment. Liprin-α4, which is required for maintenance of cell-cell contacts [36], is a hypoxia-inducible gene that was highly expressed in AMSCs under hypoxia (FC >100). TMEM45A has anti-apoptotic functions and is essential for hypoxia-induced protection against apoptosis [37]. We found that TMEM45A was overexpressed under hypoxia in RVD AMSC. Taken together, these changes in mRNA and miRNA indicate that hypoxic conditions boosted cell survival and amplified angiogenic and anti-inflammatory functions of AMSC from atherosclerotic patients.

Autophagy modulates homeostatic and cytoprotective physiological cellular functions, such as degradation of long-lived proteins, organelle turnover, adaptation to stress, extension of lifespan, and cellular development [38]. Recent studies identify crucial functions of BNIP3 and BNIP3L in hypoxia-induced autophagy and indicate that hypoxia impacts cell survival. BNIP3 family disrupts the Bcl-2/Beclin1 complex and induces autophagy, especially when Bcl-2 or Bcl-X_L_ is weakly expressed [39, 40]. In our study, BNIP3 was highly expressed under hypoxia (FC >7), while Bcl-2, Bcl-K_L_ and Beclin1 were downregulated. We suggest that hypoxia-induced autophagy may promote survival, block induction of apoptosis and reduce hypoxic cellular injury, because survival rates between cells grown under normoxia versus hypoxia were comparable, while hypoxia reduced the percentage of dead cells in most RVD patients.

**Fig. 7:**
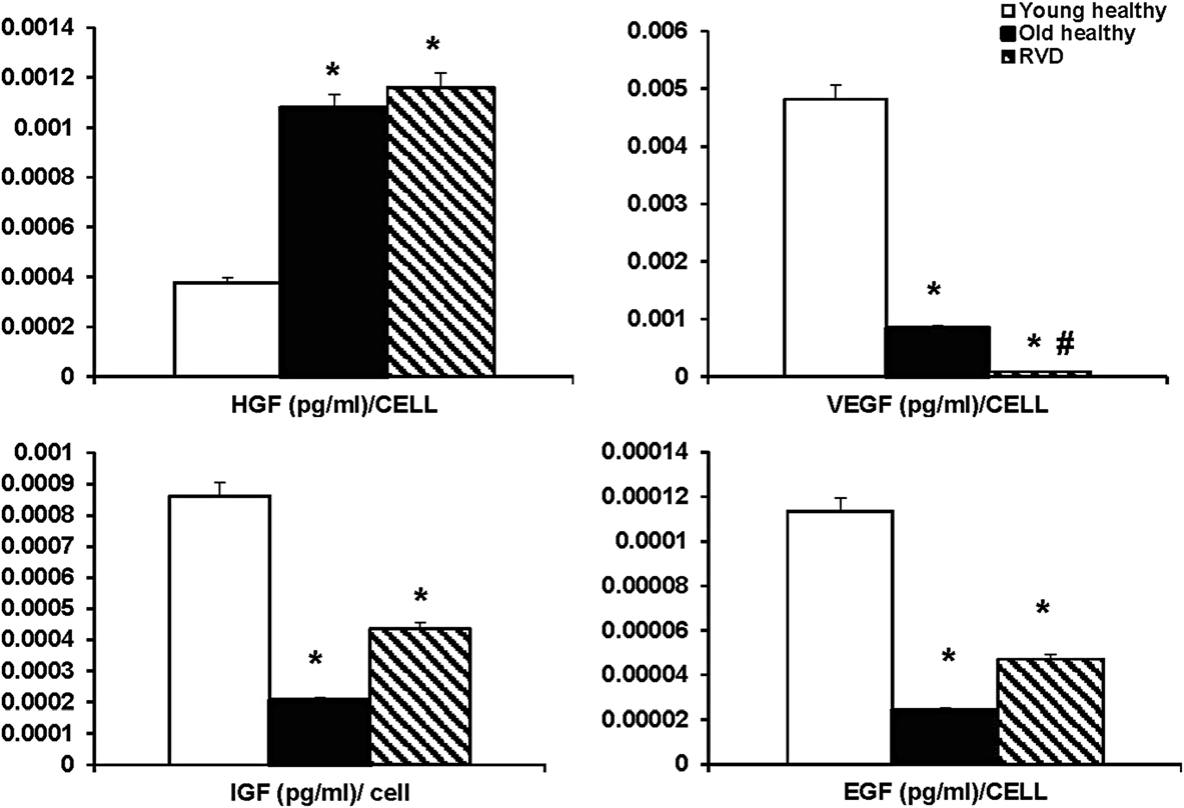
AMSCs from older healthy donors and RVD patients both showed lower secretion of VEGF, IGF and EGF compared to AMSCs from young healthy donors. These data suggest that at least some of the differential effects between healthy donor subjects and RVD are related primarily to differences in age (see text). *EGF* epidermal growth factor, *IGF* insulin-like growth factor; *HGF* hepatocyte growth factor, *RVD* renovascular disease, *VEGF* vascular endothelial growth factor

Angiogenesis potentially includes restoration of normal vascular function and structure, and the reversal of vascular senescence. Growth of new blood vessels is crucial in the treatment of RVD and other ischemic diseases [41]. Recent experimental studies indicate that renal VEGF levels are altered in pathologic situations, such as chronic and acute renal ischemia [42]. Our results indicate that AMSC from patients with RVD secreted less VEGF compared to healthy individuals in the AMSC supernatant (Fig. 7). Increased expression of angiogenic factors, including the upregulation of VEGF, has previously been shown to increase blood vessel formation in vivo [43]. Elevated expression of miR-210 induces angiogenesis and is associated with local increased VEGF levels [44]. Reduction of miR-15b and miR-16 contributes to an increase in VEGF and improve angiogenesis [45, 46]. Stanniocalcin-1 and -2 (STC 1, 2) promote angiogenic sprouting in human umbilical vein endothelial cells (HUVECs) via VEGF/VEGFR2 and angiopoietin signaling pathways [47]. Consistent with these data, we observed that hypoxia downregulated miR-101b and miR-29a and increased the expression of their target genes STC1 and STC2 in AMSCs.

Recent studies show that human miR-221 and miR-222 are important in vascular biology and exhibit marked antiangiogenic properties. They are upregulated in early atherosclerosis, causing inhibition of angiogenic recruitment of endothelial cells (ECs). These miRNAs increase endothelial dysfunction and EC apoptosis and negatively regulate VEGF [48, 49]. We showed in this study that hypoxia decreased the expression of miR-221 (FC approximately -2) and miR-222 (FC > -1.4) and increased expression of their known common target gene CDKN1.

HGF is an angiogenic growth factor that plays an important role in angiogenesis. Its level was increased in AMSCs from RVD compared to healthy donors. HGF is thought to increase the collateral vascular growth (arteriogenesis) [50], although its role is insufficient to protect vascular integrity in RVD. VEGF alone seems to affect capillary angiogenesis more efficiently than collateral growth [51]. Regulation of local HGF production under hypoxia is unclear, with most reports suggesting that it increases under hypoxia [12, 52]. However, our results indicate that HGF was downregulated under hypoxia both relative to gene expression and protein secretion. Our results are supported by the studies by Hayashi and colleagues [53] who demonstrated that hypoxic treatment of vascular cells downregulated HGF production due to decreased cAMP, consistent with their potential role in the pathophysiology of ischemic diseases.

Taken together, our results demonstrated that growing AMSCs from RVD patients under hypoxic conditions (1 % O2) altered their cellular characteristics, mRNA and protein expression and increased both survival and angiogenic potential. Oxygen concentration in medullary compartments of the kidney is normally low [30], and in kidneys with severe RVD accompanied by inflammation, the degree and extent of hypoxia increase [6, 31]. These studies suggest that functional responses of AMSC under hypoxic conditions may favorably influence their efficacy for renal repair. These effects were associated with increased secretion of several cytokines crucial for angiogenesis and survival such as VEGF.

## Conclusions

AMSCs obtained from patients with atherosclerotic RVD are different and less angiogenic from those obtained from healthy individuals. Our data support the concept that age and/or disease-related disturbances in AMSC function may be modifiable. Physiological approaches like hypoxia that normalize the biological properties of stem cells that were harvested under pathophysiological conditions (e.g., elderly RVD patients) will be important to improve cell survival and functionality, as well as permit their adaptation for novel therapies for occlusive vascular disease.

## Additional files

Additional file 1: Representative flow cytometric plots (FACS) showing that AMSCs expressed HLA-ABC, CD44, CD90, CD29, CD73, and CD105 markers, and did not express HLA-DR, CD45, CD14 or CD34 markers. (JPG 233 kb) Additional file 2: Hypoxia increased the expression of more than 460 genes. This set mRNAs encode regulators of metabolism, DNA repair, cell cycle regulation, and proteins involved in related functions associated with cell survival. (JPG 68 kb)

## Abbreviations

AMSCs: adipose-derived mesenchymal stem/stromal cells
ECs: endothelial cells
EGF: epidermal growth factor
GFR: glomerular filtration rate
HGF: hepatocyte growth factor
IGF-1: insulin-like growth factor 1
PDT: population-doubling time
RBF: renal blood flow
RPKM: reads per kilo base pair per million mapped reads
RVD: renovascular disease
VEGF: vascular endothelial growth factor

## References

[1] Saad A, Herrmann SM, Crane J, Glockner JF, McKusick MA, Misra S (2013). Stent revascularization restores cortical blood flow and reverses tissue hypoxia in atherosclerotic renal artery stenosis but fails to reverse inflammatory pathways or glomerular filtration rate. Circ Cardiovasc Interv.

[2] Textor SC, Lerman L (2010). Renovascular hypertension and ischemic nephropathy. Am J Hypertens.

[3] Chade AR, Rodriguez-Porcel M, Grande JP, Krier JD, Lerman A, Romero JC (2002). Distinct renal injury in early atherosclerosis and renovascular disease. Circulation.

[4] Saad A, Herrmann SM, Textor SC (2015). Chronic renal ischemia in humans: can cell therapy repair the kidney in occlusive renovascular disease?. Physiology (Bethesda).

[5] Eirin A, Zhu XY, Krier JD, Tang H, Jordan KL, Grande JP (2012). Adipose tissue-derived mesenchymal stem cells improve revascularization outcomes to restore renal function in swine atherosclerotic renal artery stenosis. Stem Cells.

[6] Gnecchi M, Zhang Z, Ni A, Dzau VJ (2008). Paracrine mechanisms in adult stem cell signaling and therapy. Circ Res.

[7] Singer AJ, Clark RA (1999). Cutaneous wound healing. N Engl J Med.

[8] Mohyeldin A, Garzon-Muvdi T, Quinones-Hinojosa A (2010). Oxygen in stem cell biology: a critical component of the stem cell niche. Cell Stem Cell.

[9] Brezis M, Rosen S, Silva P, Epstein FH (1984). Renal ischemia: a new perspective. Kidney Int.

[10] Hyun JS, Tran MC, Wong VW, Chung MT, Lo DD, Montoro DT (2013). Enhancing stem cell survival in vivo for tissue repair. Biotechnol Adv.

[11] De Barros S, Dehez S, Arnaud E, Barreau C, Cazavet A, Perez G (2013). Aging-related decrease of human ASC angiogenic potential is reversed by hypoxia preconditioning through ROS production. Mol Ther.

[12] Rosova I, Dao M, Capoccia B, Link D, Nolta JA (2008). Hypoxic preconditioning results in increased motility and improved therapeutic potential of human mesenchymal stem cells. Stem Cells.

[13] Tang YL, Zhu W, Cheng M, Chen L, Zhang J, Sun T (2009). Hypoxic preconditioning enhances the benefit of cardiac progenitor cell therapy for treatment of myocardial infarction by inducing CXCR4 expression. Circ Res.

[14] Wisel S, Khan M, Kuppusamy ML, Mohan IK, Chacko SM, Rivera BK (2009). Pharmacological preconditioning of mesenchymal stem cells with trimetazidine (1-[2,3,4-trimethoxybenzyl]piperazine) protects hypoxic cells against oxidative stress and enhances recovery of myocardial function in infarcted heart through Bcl-2 expression. J Pharmacol Exp Ther.

[15] Crespo-Diaz R, Behfar A, Butler GW, Padley DJ, Sarr MG, Bartunek J (2011). Platelet lysate consisting of a natural repair proteome supports human mesenchymal stem cell proliferation and chromosomal stability. Cell Transplant.

[16] Dudakovic A, Camilleri E, Riester SM, Lewallen EA, Kvasha S, Chen X (2014). High-resolution molecular validation of self-renewal and spontaneous differentiation in clinical-grade adipose-tissue derived human mesenchymal stem cells. J Cell Biochem.

[17] Sun Z, Evans J, Bhagwate A, Middha S, Bockol M, Yan H (2014). CAP-miRSeq: a comprehensive analysis pipeline for microRNA sequencing data. BMC Genomics..

[18] Robinson MD, McCarthy DJ, Smyth GK (2010). edgeR: a Bioconductor package for differential expression analysis of digital gene expression data. Bioinformatics.

[19] Wang X (2008). miRDB: a microRNA target prediction and functional annotation database with a wiki interface. RNA.

[20] Strober W. Trypan Blue Exclusion Test of Cell Viability. Curr Protoc Immunol. 2001;21:3B:A.3B.1–A.3B.2. 10.1002/0471142735.ima03bs21 18432654

[21] Wang H, Cai S, Ernstberger A, Bailey BJ, Wang MZ, Cai W (2013). Temozolomide-mediated DNA methylation in human myeloid precursor cells: differential involvement of intrinsic and extrinsic apoptotic pathways. Clin Cancer Res.

[22] Fragkos M, Jurvansuu J, Beard P (2009). H2AX is required for cell cycle arrest via the p53/p21 pathway. Mol Cell Biol.

[23] Anderson KR, Singer RA, Balderes DA, Hernandez-Lagunas L, Johnson CW, Artinger KB (2011). The L6 domain tetraspanin Tm4sf4 regulates endocrine pancreas differentiation and directed cell migration. Development.

[24] Sun CY, She XM, Qin Y, Chu ZB, Chen L, Ai LS (2013). miR-15a and miR-16 affect the angiogenesis of multiple myeloma by targeting VEGF. Carcinogenesis.

[25] Wang Y, Fan H, Zhao G, Liu D, Du L, Wang Z (2012). miR-16 inhibits the proliferation and angiogenesis-regulating potential of mesenchymal stem cells in severe pre-eclampsia. FEBS J.

[26] Liu W, Gong Q, Ling J, Zhang W, Liu Z, Quan J (2014). Role of miR-424 on angiogenic potential in human dental pulp cells. J Endod.

[27] Medina R, Zaidi SK, Liu CG, Stein JL, van Wijnen AJ, Croce CM (2008). MicroRNAs 221 and 222 bypass quiescence and compromise cell survival. Cancer Res.

[28] Chan SY, Loscalzo J (2010). MicroRNA-210: a unique and pleiotropic hypoxamir. Cell Cycle.

[29] Kim JH, Park SG, Song SY, Kim JK, Sung JH (2013). Reactive oxygen species-responsive miR-210 regulates proliferation and migration of adipose-derived stem cells via PTPN2. Cell Death Dis..

[30] Chen F, Hu SJ (2012). Effect of microRNA-34a in cell cycle, differentiation, and apoptosis: a review. J Biochem Mol Toxicol.

[31] Park H, Pak HJ, Yang DY, Kim YH, Choi WJ, Park SJ (2015). miR-34a inhibits differentiation of human adipose tissue-derived stem cells by regulating cell cycle and senescence induction. Differentiation.

[32] Xu Q, Seeger FH, Castillo J, Iekushi K, Boon RA, Farcas R (2012). Micro-RNA-34a contributes to the impaired function of bone marrow-derived mononuclear cells from patients with cardiovascular disease. J Am Coll Cardiol.

[33] Zhu S, Deng S, Ma Q, Zhang T, Jia C, Zhuo D (2013). MicroRNA-10A* and MicroRNA-21 modulate endothelial progenitor cell senescence via suppressing high-mobility group A2. Circ Res.

[34] Price JG, Idoyaga J, Salmon H, Hogstad B, Bigarella CL, Ghaffari S (2015). CDKN1A regulates Langerhans cell survival and promotes Treg cell generation upon exposure to ionizing irradiation. Nat Immunol.

[35] Fornari F, Gramantieri L, Ferracin M, Veronese A, Sabbioni S, Calin GA (2008). MiR-221 controls CDKN1C/p57 and CDKN1B/p27 expression in human hepatocellular carcinoma. Oncogene.

[36] Mattauch S, Sachs M, Behrens J (2010). Liprin-alpha4 is a new hypoxia-inducible target gene required for maintenance of cell-cell contacts. Exp Cell Res.

[37] Flamant L, Roegiers E, Pierre M, Hayez A, Sterpin C, De Backer O (2012). TMEM45A is essential for hypoxia-induced chemoresistance in breast and liver cancer cells. BMC Cancer..

[38] Mazure NM, Pouyssegur J (2010). Hypoxia-induced autophagy: cell death or cell survival?. Curr Opin Cell Biol.

[39] Bellot G, Garcia-Medina R, Gounon P, Chiche J, Roux D, Pouyssegur J (2009). Hypoxia-induced autophagy is mediated through hypoxia-inducible factor induction of BNIP3 and BNIP3L via their BH3 domains. Mol Cell Biol.

[40] Lin J, Zheng Z, Li Y, Yu W, Zhong W, Tian S (2009). A novel Bcl-XL inhibitor Z36 that induces autophagic cell death in Hela cells. Autophagy.

[41] Chade AR (2016). Vascular endothelial growth factor therapy for the kidney: are we there yet?. J Am Soc Nephrol.

[42] Basile DP, Fredrich K, Chelladurai B, Leonard EC, Parrish AR (2008). Renal ischemia reperfusion inhibits VEGF expression and induces ADAMTS-1, a novel VEGF inhibitor. Am J Physiol Renal Physiol.

[43] Liu LX, Lu H, Luo Y, Date T, Belanger AJ, Vincent KA (2002). Stabilization of vascular endothelial growth factor mRNA by hypoxia-inducible factor 1. Biochem Biophys Res Commun.

[44] Li M, Tan J, Miao Y, Lei P, Zhang Q (2015). The dual role of autophagy under hypoxia-involvement of interaction between autophagy and apoptosis. Apoptosis.

[45] Hua Z, Lv Q, Ye W, Wong CK, Cai G, Gu D (2006). MiRNA-directed regulation of VEGF and other angiogenic factors under hypoxia. PLoS One..

[46] Yin KJ, Olsen K, Hamblin M, Zhang J, Schwendeman SP, Chen YE (2012). Vascular endothelial cell-specific microRNA-15a inhibits angiogenesis in hindlimb ischemia. J Biol Chem.

[47] Law AY, Wong CK (2013). Stanniocalcin-1 and -2 promote angiogenic sprouting in HUVECs via VEGF/VEGFR2 and angiopoietin signaling pathways. Mol Cell Endocrinol.

[48] Chistiakov DA, Sobenin IA, Orekhov AN, Bobryshev YV (2015). Human miR-221/222 in physiological and atherosclerotic vascular remodeling. Biomed Res Int..

[49] Khella HW, Butz H, Ding Q, Rotondo F, Evans KR, Kupchak P (2015). miR-221/222 are involved in response to sunitinib treatment in metastatic renal cell carcinoma. Mol Ther.

[50] Cao R, Brakenhielm E, Pawliuk R, Wariaro D, Post MJ, Wahlberg E (2003). Angiogenic synergism, vascular stability and improvement of hind-limb ischemia by a combination of PDGF-BB and FGF-2. Nat Med.

[51] Helisch A, Schaper W (2003). Arteriogenesis: the development and growth of collateral arteries. Microcirculation.

[52] Kitajima Y, Ide T, Ohtsuka T, Miyazaki K (2008). Induction of hepatocyte growth factor activator gene expression under hypoxia activates the hepatocyte growth factor/c-Met system via hypoxia inducible factor-1 in pancreatic cancer. Cancer Sci.

[53] Hayashi S, Morishita R, Nakamura S, Yamamoto K, Moriguchi A, Nagano T (1999). Potential role of hepatocyte growth factor, a novel angiogenic growth factor, in peripheral arterial disease: downregulation of HGF in response to hypoxia in vascular cells. Circulation.

