# Supplementary figures and images for: Adipose-derived mesenchymal stem cells from patients with atherosclerotic renovascular disease have increased DNA damage and reduced angiogenesis that can be modified by hypoxia
Source: Stem Cell Res Ther. 2016 Sep 9;7(1):128. doi: 10.1186/s13287-016-0389-x (PMC5016873; doi:10.1186/s13287-016-0389-x)

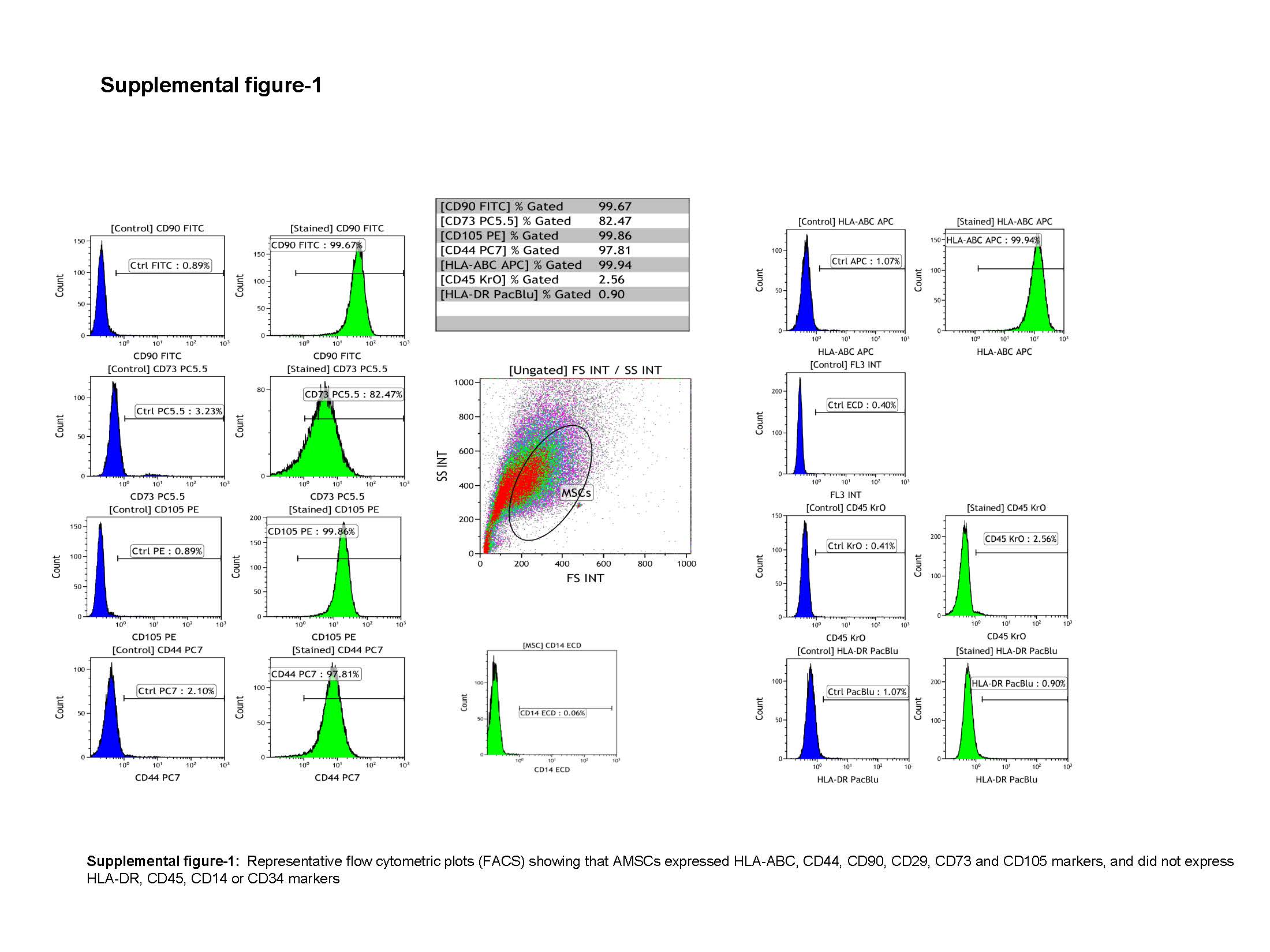

Supplement: Additional file 1: — Representative flow cytometric plots (FACS) showing that AMSCs expressed HLA-ABC, CD44, CD90, CD29, CD73, and CD105 markers, and did not express HLA-DR, CD45, CD14 or CD34 markers. (JPG 233 kb) [file 13287_2016_389_MOESM1_ESM.jpg]

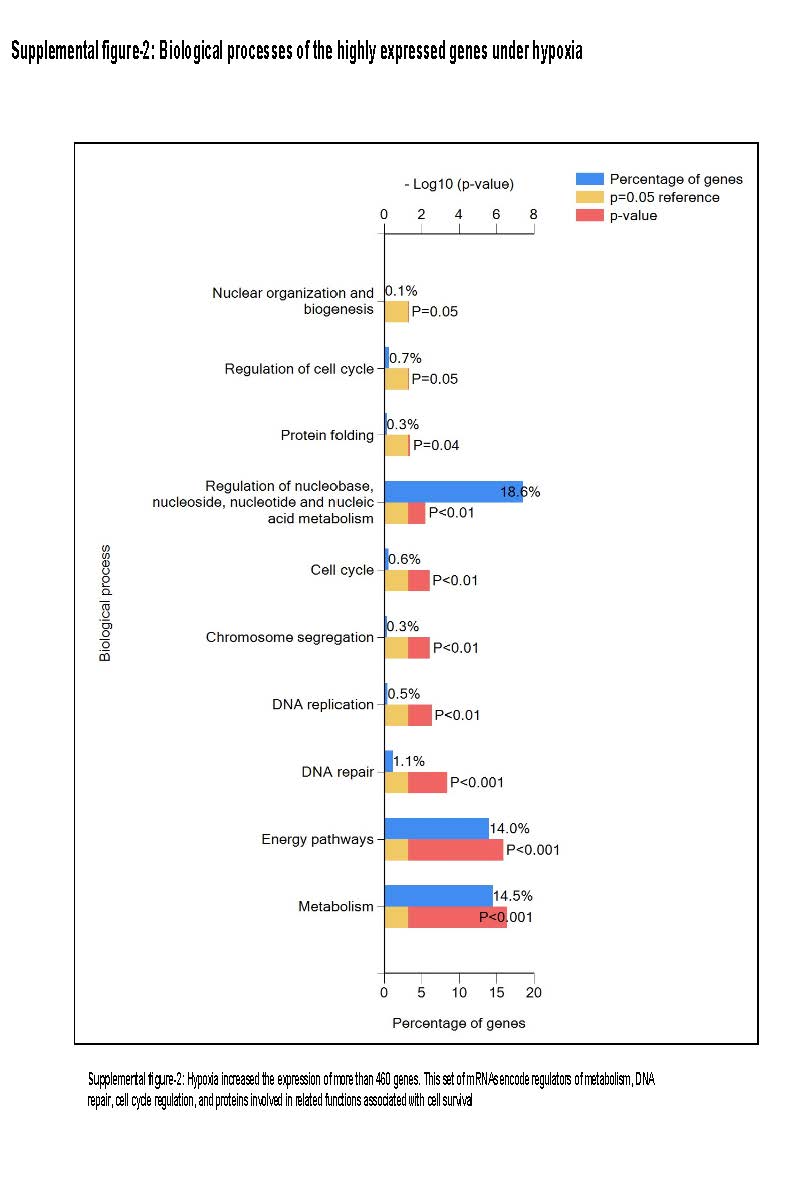

Supplement: Additional file 2: — Hypoxia increased the expression of more than 460 genes. This set mRNAs encode regulators of metabolism, DNA repair, cell cycle regulation, and proteins involved in related functions associated with cell survival. (JPG 68 kb) [file 13287_2016_389_MOESM2_ESM.jpg]
